# Arbidol (Umifenovir): A Broad-Spectrum Antiviral Drug That Inhibits Medically Important Arthropod-Borne Flaviviruses

**DOI:** 10.3390/v10040184

**Published:** 2018-04-10

**Authors:** Jan Haviernik, Michal Štefánik, Martina Fojtíková, Sabrina Kali, Noël Tordo, Ivo Rudolf, Zdeněk Hubálek, Luděk Eyer, Daniel Ruzek

**Affiliations:** 1 Department of Virology, Veterinary Research Institute, Hudcova 70, CZ-62100 Brno, Czech Republic; haviernik@vri.cz (J.H.); stefanik@vri.cz (M.Š.); fojtikova@vri.cz (M.F.); eyer@vri.cz (L.E.); 2 Unit Antiviral Strategies, Institut Pasteur, 25 Dr. Roux, 75724 Paris CEDEX 15, France; sabrina.kali@pasteur.fr (S.K.); ntordo@pasteur.fr (N.T.); 3 Insitut Pasteur de Guinée, route de Donka, Conakry, Guinea; 4 Institute of Vertebrate Biology, Czech Academy of Sciences, Kvetna 8, CZ-60365 Brno, Czech Republic; rudolf@ivb.cz (I.R.); zhubalek@brno.cas.cz (Z.H.); 5 Institute of Parasitology, Biology Centre of the Czech Academy of Sciences, Branisovska 31, CZ-37005 Ceske Budejovice, Czech Republic

**Keywords:** flavivirus, arbidol, umifenovir, antiviral activity, cytotoxicity, cell-type dependent antiviral effect

## Abstract

Arthropod-borne flaviviruses are human pathogens of global medical importance, against which no effective small molecule-based antiviral therapy has currently been reported. Arbidol (umifenovir) is a broad-spectrum antiviral compound approved in Russia and China for prophylaxis and treatment of influenza. This compound shows activities against numerous DNA and RNA viruses. The mode of action is based predominantly on impairment of critical steps in virus-cell interactions. Here we demonstrate that arbidol possesses micromolar-level anti-viral effects (EC_50_ values ranging from 10.57 ± 0.74 to 19.16 ± 0.29 µM) in Vero cells infected with Zika virus, West Nile virus, and tick-borne encephalitis virus, three medically important representatives of the arthropod-borne flaviviruses. Interestingly, no antiviral effects of arbidol are observed in virus infected porcine stable kidney cells (PS), human neuroblastoma cells (UKF-NB-4), and human hepatoma cells (Huh-7 cells) indicating that the antiviral effect of arbidol is strongly cell-type dependent. Arbidol shows increasing cytotoxicity when tested in various cell lines, in the order: Huh-7 < HBCA < PS < UKF-NB-4 < Vero with CC_50_ values ranging from 18.69 ± 0.1 to 89.72 ± 0.19 µM. Antiviral activities and acceptable cytotoxicity profiles suggest that arbidol could be a promising candidate for further investigation as a potential therapeutic agent in selective treatment of flaviviral infections.

## 1. Introduction

Arthropod-borne flaviviruses (genus *Flavivirus*, family *Flaviviridae*) include human pathogens of global medical importance such as dengue virus (DENV), Yellow fever virus (YFV), West Nile virus (WNV), Japanese encephalitis virus (JEV), Zika virus (ZIKV), Kyasanur Forest disease virus (KFDV), Omsk hemorrhagic fever virus (OHFV) and tick-borne encephalitis virus (TBEV). These viruses are causative of many serious diseases with a broad spectrum of clinical symptoms ranging from near asymptomatic or mild flu-like infections through neurological diseases (WNV, TBEV) to viscerotropic (DENV), hemorrhagic (KFDV, OHFV) or even terratogenic manifestations (ZIKV) [1,2]. Up to 200 million new cases of infection caused by arthropod-borne flaviviruses are reported annually [1]. These viruses can be widespread, as exemplified by the epidemiological outbreaks of WNV infection across North America, Mexico, South America, and the Caribbean during 1999–2002 [3,4], or ZIKV infection in Oceania and Latin America during 2014–2016 [5]. At present, there is no effective antiviral therapy directed against these viruses, and therefore, search for small molecule-based inhibitors represents an unmet medical need.

Arbidol, also known as umifenovir, is a broad-spectrum antiviral compound developed at the Russian Research Chemical and Pharmaceutical Institute about 25 years ago [6] and licensed in Russia and China for the prophylaxis and treatment of human influenza A and B infections, plus post-influenza complications [7]. Subsequently, arbidol was shown to be active against numerous DNA/RNA and enveloped/non-enveloped viruses [8]. The predominant mode of action of arbidol is based on its intercalation into membrane lipids leading to the inhibition of membrane fusion between virus particles and plasma membranes, and between virus particles and the membranes of endosomes [9]. In influenza virus, arbidol was observed to interact with virus hemagglutinin (HA), causing an increase in HA stability thereby preventing the pH-induced transition of HA into its a functional fusogenic state [10]. In the case of hepatitis C virus (HCV) arbidol interacts with HCV envelope protein to cause various degrees of inhibition to critical membrane fusion events [11,12]. Alternatively, arbidol may also be immunomodulatory, and as such be capable of interferon induction and/or macrophage activation [13]. Due to such broad-spectrum antiviral activities, arbidol represents a promising candidate for treatment of viral infections in humans.

Accordingly, we describe here using standardized in vitro assay systems the cytotoxicities and antiviral activities of arbidol against three representative flaviviruses; ZIKV and WNV as emerging mosquito-borne pathogens, and TBEV as an important tick-borne pathogen. Since antiviral compounds are extensively inactivated/metabolized in the intracellular environment [14,15], different cell lines were utilized to assess simultaneously both the antiviral and corresponding cytotoxic effects of arbidol.

## 2. Material and Methods

**Figure 1:**
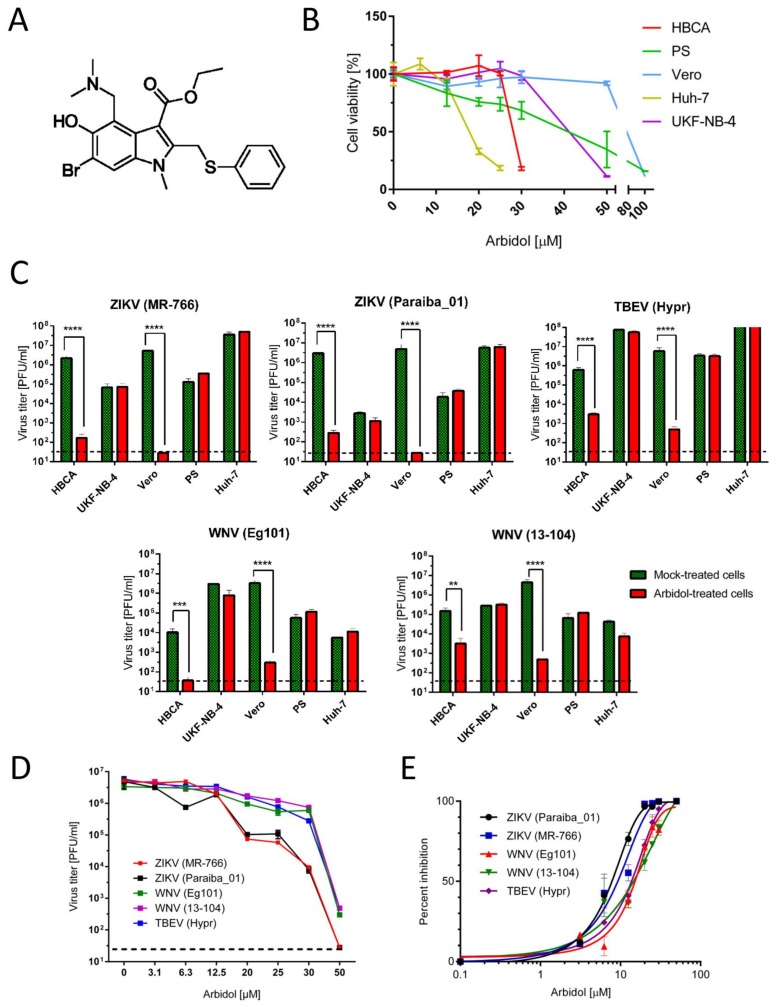
(**A**) Structure of arbidol. (**B**) Cytotoxicities of arbidol with Huh-7, PS, UKF-NB-4, HBCA, and Vero cells within the compound concentration ranges 0–100 μM, 48 h post infection. (**C**) Antiviral effects of arbidol against ZIKV, WNV and TBEV infection in different cell lines. Given differential arbidol cytotoxicities with respect to different cell lines, indicated cell lines were treated with different maximum concentrations of arbidol (12.5 µM for Huh-7, 25 µM for HBCA and PS, 30 µM for UKF-NB-4, and 50 µM for Vero) 24 h prior to virus infection. Culture supernatants were then collected 48 h post infection and individual viral titers were determined by plaque assay. (**D**) Dose-dependent effects of arbidol on virus titers 48 h post infection in Vero cells. The horizontal dashed line indicates the minimum detectable threshold of 1.44 log_10_ PFU/mL. (**E**) Inhibition of indicated flaviviruses in the presence of a serial dilution of arbidol. Data from two (**C**) or three (**B**–**E**) independent experiments done in triplicates. ** *p* < 0.01; *** *p* < 0.001; **** *p* < 0.0001.

Arbidol (ethyl-6-bromo-4-[(dimethylamino)methyl]-5-hydroxy-1-methyl-2-[(phenylthio)methyl]-indole-3-carboxylate hydrochloride monohydrate) (Figure 1A) was obtained from Sigma-Aldrich (St. Louis, MO, USA), solubilized in 100% DMSO to yield 10 mM stock solution. The following viral strains/isolates were used in this study: ZIKV (MR766, a representative of the African ZIKV lineage; Paraiba_01, a member of the Asian ZIKV lineage), WNV (strains Eg101 and 13-104), and TBEV (strain Hypr, a typical representative of the West European TBEV subtype). As ZIKV, WNV and TBEV are neurotropic viruses, cell lines of both neuronal as well as extraneural origin were selected for antiviral screens, virus multiplication and plaque assays. Porcine kidney stable (PS) cells [16] were cultured in Leibovitz (L-15) medium, human brain cortical astrocytes (HBCA) (ScienCell, Carlsbad, CA, USA) were cultivated in Astrocyte medium (Thermo Fisher Scientific, Weltham, MA, USA), human neuroblastoma UKF-NB-4 cells [17] were cultured in Iscove’s modified Dulbecco’s medium (IMDM), Vero cells (ATCC CCL-81, African Green Monkey, adult kidney, epithelial) and human hepatocellular carcinoma cells (Huh-7) were grown in Dulbecco’s modified Eagle’s medium (DMEM). The media were supplemented with 3% (L-15), 6% (Astrocyte medium), or 10% (IMDM and DMEM) newborn calf serum and a 1% mixture of antibiotics and antimycotics and 1% glutamine (Sigma-Aldrich, Prague, Czech Republic).

The compound cytotoxicity was determined in terms of cell viability using the Cell Counting Kit-8 (Dojindo Molecular Technologies, Munich, Germany) according to the manufacturer’s instructions and expressed as the 50% cytotoxic concentration (CC_50_), which represents the concentration of compound that reduced cell viability by 50%. A viral titer reduction assay was performed to determine the flavivirus sensitivity to arbidol in cell culture. Host cells were seeded in 96-well plates (approximately 2 × 10^4^ cells per well) and incubated for 24 h at 37 °C to form a confluent monolayer. The medium was then aspirated from the wells and replaced with 200 µL of fresh medium containing 0–12.5 µM (for Huh-7), 0–25 µM (for HBCA and PS), 0–30 µM (for UKF-NB-4), or 0–50 µM (for Vero) of arbidol and incubated for 24 h (the concentration ranges were based on different cytotoxicity effects of arbidol for individual cell lines, as described below). The medium was then removed from wells and replaced with fresh medium containing arbidol and appropriate virus strain at a multiplicity of infection (MOI) of 0.1. After 2 h incubation, the medium was replaced with fresh medium containing arbidol and incubated for 48 h at 37 °C. Then, the supernatant medium was collected and viral titers were determined by plaque assays, expressed as PFU/mL [18] and used for construction of dose-response and inhibition curves and for estimation the 50% effective concentration (EC_50_). For construction of growth curves, Vero cells were infected as described above and treated with arbidol at a concentration of 50 µM. Supernatant media were collected every six hours until 48 h p.i. Viral titers in supernatants were determined using plaque assay. In all experiments, DMSO was added to virus-infected cells as a negative control at a concentration corresponding to a dilution of the initial arbidol-DMSO stock. A cell-based flavivirus immunostaining assay was performed to measure the arbidol-induced inhibition of viral surface antigen (E protein) expression, as previously described [19].

Differences in viral titer between groups were evaluated by unpaired parametric two-tailed *t*-test using GraphPad Prism 7 (GraphPad Software, Inc., La Jolla, CA, USA), version 7.04. Differences with *p* < 0.05 were considered to be statistically significant.

## 3. Results and Discussion

We initially determined arbidol cytotoxity profiles for Huh-7, Vero, PS, UKF-NB-4, and HBCA cells. As is apparent (Figure 1B), arbidol was differentially cytotoxic. Lowest cytotoxicites were observed with Vero cells (CC_50_ = 89.72 ± 0.19 µM). This level of cytotoxicity was about 5 times lower than observed with Huh-7 cells (CC_50_ = 18.69 ± 0.1 µM) that were the most susceptible cells studied. Experiments with other cell types, namely PS, UKF-NB-4, and HBCA gave rise to CC_50_ values ranging from 24.78 ± 0.01 to 46.99 ± 0.1 µM (Table 1). The variable cytotoxic effects of arbidol may be related to its broad-spectrum activity impairing crucial cellular metabolic pathways or critical steps in virus-cell interactions [11,20]. This contrasts with other antiviral drugs that preferentially target viral proteins, such as nucleoside inhibitors of viral polymerases, for which the CC_50_ values usually do not exceed typically 100 µM [21].

The antiviral effects of arbidol against two ZIKV strains (MR766 and Paraiba_01) were evident in both Vero and HBCA cells 48 h after infection. Whereas in HBCA the highest arbidol concentration tested (25 µM) caused a reduction in ZIKV titer of about 10^4^-fold compared to the situation in mock-treated cells, in Vero cells viral replication was inhibited completely at 50 µM. ZIKV replication was not inhibited in UKF-NB-4, PS, and Huh-7 cells, suggesting that the antiviral effects of arbidol are strongly cell type-dependent (Figure 1C). This phenomenon has been described previously with many other antiviral compounds and is thought to arise from differences in the expression levels of intracellular enzymes/proteins involved in metabolism and transport [21]. These differences apparently ensure that the same inhibitor should exhibit different EC_50_ values according to the cell line employed [22,23]. For example, sofosbuvir was found to be differentially active against ZIKV infection depending on the cultivated cells used, owing to differences in intracellular processing [24].

**Figure 2:**
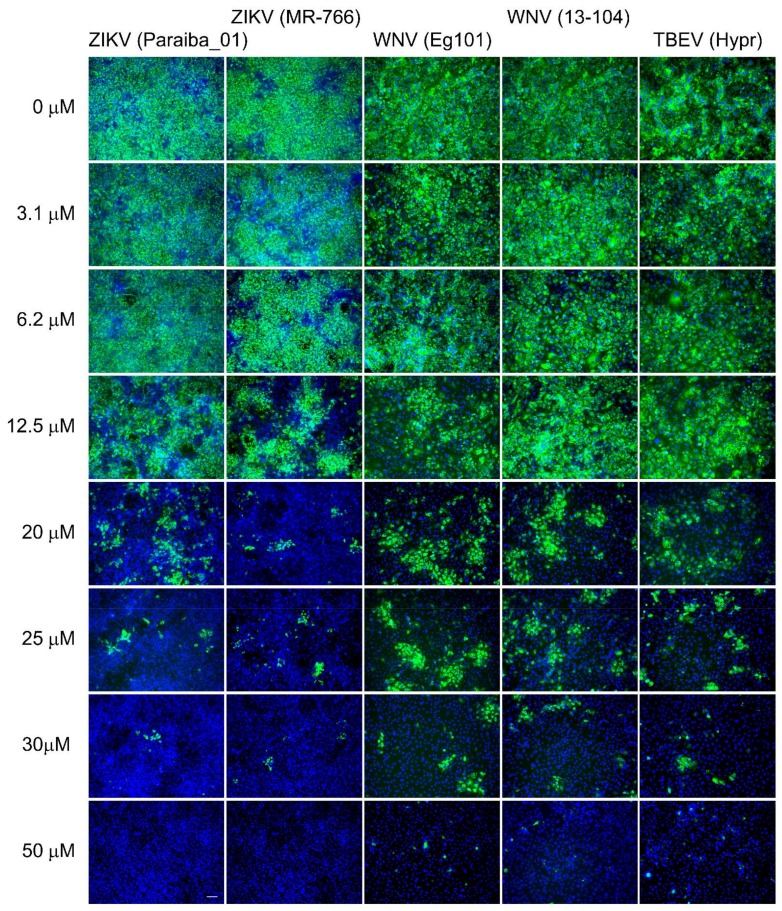
Inhibition of flaviviral surface E antigen expression by arbidol. Vero cells infected with virus were fixed on slides 48 h after infection and stained with flavivirus-specific antibody labeled with FITC (green) and counterstained with DAPI (blue). Scale bar, 50 µm.

Based on these results, we further evaluated the inhibitory potential of arbitol against viral infections in Vero cells in particular. For instance, the EC_50_ values for ZIKV infections in Vero cells were found to be 12.09 ± 0.77 and 10.57 ± 0.74 µM respectively for MR766 and Paraiba_01 strains (Figure 1D,E, Table 2). The antiviral effects of arbidol were further confirmed by immunofluorescence staining. Using this method, a dose-dependent inhibition of ZIKV surface E antigen expression was observed in Vero cells (Figure 2). The observed anti-ZIKV properties are comparable with those of previously reported small-molecules, such as nucleoside analogues, that are known to exert anti-ZIKV inhibitions in the micromolar range (0.2 to 22 µM) according to the cell-based assay systems used [25,26,27,28].

Arbidol also showed significant in vitro antiviral efficacies when tested against two strains of WNV (Eg101 and 13-104). As with ZIKV, the anti-WNV effects of arbidol were most obvious when incubated at 25 and 50 µM with WNV-infected HBCA and Vero cells for 48 h. By contrast, there were little or no antiviral effects observed in UKF-NB-4, PS, and Huh-7 virus infected cells (Figure 1C). EC_50_ values in Vero cells were found to be 18.78 ± 0.21 and 19.16 ± 0.29 µM for Eg101 and 13-104, respectively, values that were slightly higher (about two-times) in comparison to values found with ZIKV (Figure 1D,E, Table 2). This observed arbidol-mediated decrease in WNV titer was strongly correlated with dose-dependent inhibition of viral surface E antigen expression in the compound-treated Vero cell culture (Figure 2).

Finally, TBEV (strain Hypr) infections in HBCA and Vero cells (Figure 1C), also proved susceptible to arbidol-mediated inhibition in dose-dependent manners, as shown by EC_50_ values of 18.67 ± 0.15 µM (Figure 1D,E, Table 2). However, when arbidol was introduced at the highest appropriate concentrations (25 µM for HBCA and 50 µM for Vero cells) inhibition of TBEV replication remained incomplete in cell culture, although the viral titer was reduced by 10^3^-fold compared with non-treated cells (Figure 1C and Figure 2). 

In Vero cells, arbidol administration was found to inhibit viral replication in all cases. In fact, a rapid reduction of viral titer was observed within the first 6 h p.i.; then the titer remained low (or even below detection limit) until the end of the experiment. In untreated cells, there was a peak in virus production between 12 and 30 h p.i. reaching a maximum of 10^6.5^ to 10^8^ PFU/mL (Supplementary Figure S1).

Our data agree with numerous reports of in vitro arbidol-mediated inhibition of other viruses of medical interest. Arbidol inhibits replication of various subtypes of human influenza A and B viruses with EC_50_ values ranging from 3 to 9 µg/mL [29]. Arbidol is also known to inhibit Chikungunya virus replication in Vero cells or in primary human fibroblasts with EC_50_ values < 10 µg/mL [30], plus inhibit Crimean-Congo hemorrhagic fever virus replication with an EC_50_ value of 2.8 µg/mL [31]. Furthermore, treatment of human hepatocellular carcinoma cells Huh 7.5.1 with 15 µM of arbidol for 24 h to 48 h, led to inhibition of HCV replication by up to 10^3^-fold [11]. Overall, arbidol exhibits a broad range antiviral effects against respiratory syncytial virus, hepatitis B virus, adenovirus, parainfluezna virus, avian coronavirus, coxackie B3 virus, and hantaan virus indicating broad-spectrum antiviral activities [11,32,33]. Besides in vitro studies, arbidol also exerts substantial antiviral effects in various animal models of infection and has been used with effect in clinical trials for the prevention and treatment of influenza [33].

## 4. Conclusions

In conclusion, we have demonstrated that arbidol has substantial antiviral activities against ZIKV, WNV and TBEV in vitro with EC_50_ values ranging from 10.57 ± 0.74 to 19.16 ± 0.29 µM. The observed antiviral effects were strongly cell-type dependent being substantial only in HBCA and Vero cells. Arguably, such cell line variability results from differences in arbitol up-take or metabolic processing by the different individual cell lines concerned. Arbidol-mediated cytotoxicities are also cell line dependent, with Huh-7 cells being the most susceptible and Vero cells the least. Our data broaden the possibility for future in vivo testing of arbidol in animal models of flavivirus infection.

## Figures and Tables

**Table 1:** Cytotoxicity of arbidol for various cell lines of neurone or extraneural origin.

Cell Line	CC_50_ (µM) ^a^
Human brain cortical astrocytes (HBCA)	24.78 ± 0.01
Human neuroblastoma UKF-NB-4 cells	46.99 ± 0.10
Vero cells	89.72 ± 0.19
Human hepatocarcinoma cells (Huh-7)	18.69 ± 0.10
Porcine kidney stable cells (PS)	46.81 ± 1.65

^a^ Determined from three independent experiments performed in triplicate. Calculated as a 50% reduction in cell viability using the Reed-Muench method.

**Table 2:** Anti-flaviviral activity and cytotoxicity characteristics of arbidol in Vero cells.

Virus	Strain	EC_50_ (µM) ^a,b^	CC_50_ (µM) ^a,c^	SI (CC_50_/EC_50_)
ZIKV	MR-766	12.09 ± 0.77	89.72 ± 0.19	7.42
	Paraiba_01	10.57 ± 0.74		8.49
WNV	Eg101	18.78 ± 0.21		4.78
	13-104	19.16 ± 0.29		4.68
TBEV	Hypr	18.67 ± 0.15		4.81

^a^ Determined from three independent experiments performed in triplicate. ^b^ Calculated as a 50% reduction in viral titers using the Reed-Muench method. ^c^ CC_50_ value determined for Vero cells. SI, selectivity index.

## Supplementary Materials

The following are available online at http://www.mdpi.com/1999-4915/10/4/184/s1, Figure S1: Growth curves of West Nile virus (WNV), Zika virus (ZIKV), and tick-borne encephalitis virus (TBEV) in Vero cells treated or mock-treated with arbidol.
