# Supplementary material for: Arbidol (Umifenovir): A Broad-Spectrum Antiviral Drug That Inhibits Medically Important Arthropod-Borne Flaviviruses
Source: Viruses. 2018 Apr 10;10(4):184. doi: 10.3390/v10040184 (PMC5923478; doi:10.3390/v10040184)
Supplement: Supplementary file 1 [file viruses-10-00184-s001.pdf]

Supplementary Figure 1

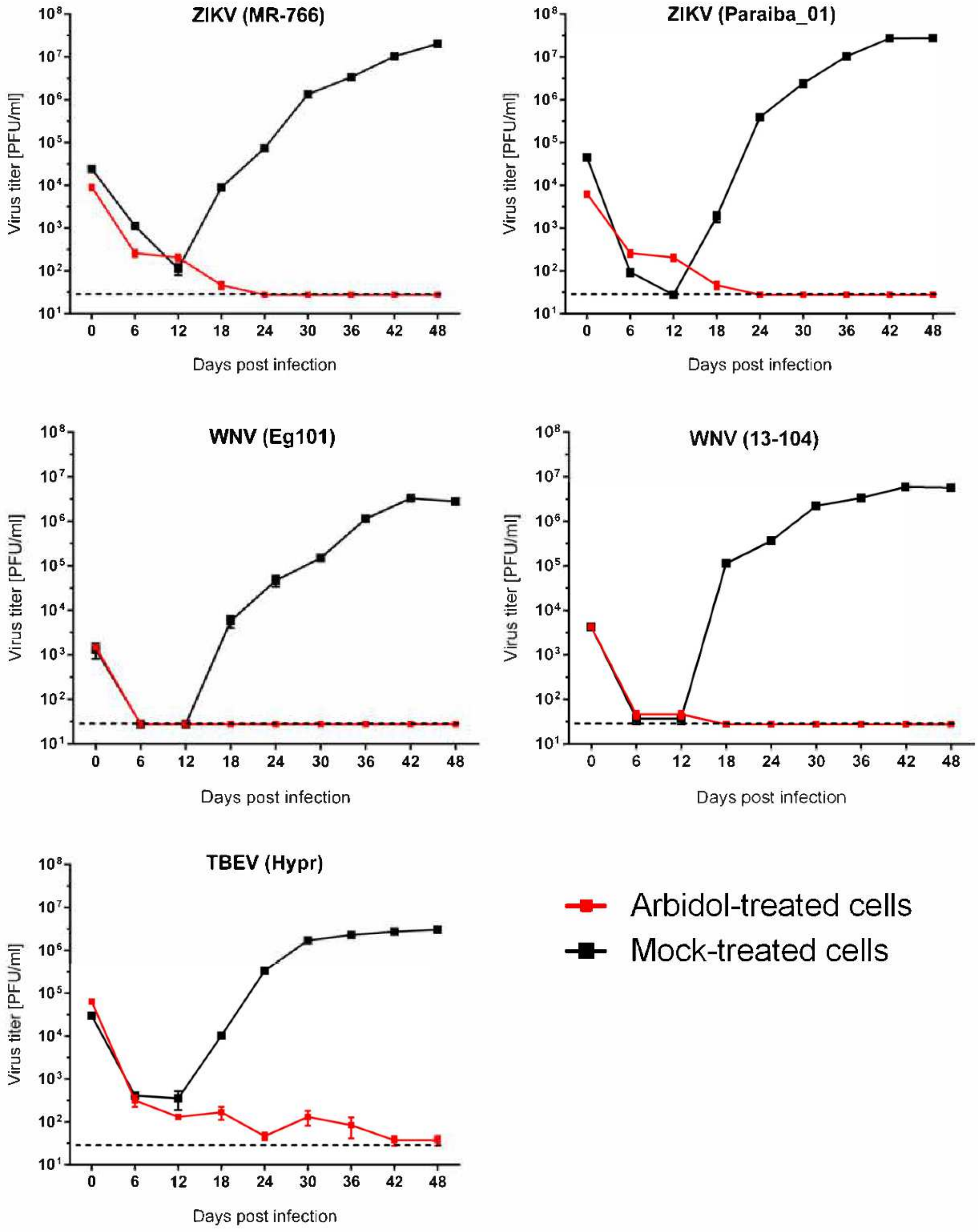

Growth curves of West Nile virus (WNV), Zika virus (ZIKV), and tick-borne encephalitis virus (TBEV) in Vero cells treated or mock-treated with arbidol. Supernatant media were collected every 6 hours and viral titres were determined by plaque assay.

Vero cells were seeded in 96-well plates (approximately 2×10^4 cells per well), and incubated for 24 h at 37 °C to form a confluent monolayer. The medium was then aspirated from the wells and replaced with 200 µl of fresh medium containing 0 or 50 µM of arbidol and incubated for 24 hours. The medium was then removed from wells and replaced with fresh medium containing arbidol and appropriate virus strain at a multiplicity of infection of 0.1. After 2 h incubation, the medium was replaced with fresh medium containing arbidol. Supernatant media were collected every six hours until 48 hours p.i. Viral titres in supernatants were determined using plaque assay.
